# Mitochondrial complex deficiency by novel compound heterozygous *TMEM70* variants and correlation with developmental delay, undescended testicle, and left ventricular noncompaction in a Japanese patient: A case report

**DOI:** 10.1002/ccr3.2050

**Published:** 2019-02-07

**Authors:** Keiichi Hirono, Fukiko Ichida, Natsuhito Nishio, Minako Ogawa‐Tominaga, Takuya Fushimi, Rene′ G. Feichtinger, Johannes A. Mayr, Masakazu Kohda, Yoshihito Kishita, Yasushi Okazaki, Akira Ohtake, Kei Murayama

**Affiliations:** ^1^ Department of Pediatrics Graduate School of Medicine University of Toyama Toyama Japan; ^2^ Department of Pediatrics Ishikawa Prefectural Central Hospital Kanazawa Japan; ^3^ Department of Metabolism Center for Medical Genetics Chiba Children's Hospital Midori‐ku Chiba Japan; ^4^ Department of Pediatrics University Hospital Salzburg Paracelsus Medical University Salzburg Austria; ^5^ Intractable Disease Research Center Graduate School of Medicine Juntendo University Tokyo Japan; ^6^ Faculty of Medicine Department of Pediatrics Saitama Medical University Saitama Japan

**Keywords:** cardiomyopathy, mitochondrial disease, *TMEM70*

## Abstract

We identified novel compound heterozygous *TMEM70* variants in a Japanese patient who had hyperlactacidemia, metabolic acidosis, hyperalaninemia, developmental delay, undescended testicle, and left ventricular noncompaction. The urinary organic acids profile revealed elevated levels of 3‐MGA, and BN‐PAGE/Western blotting analysis and ETC. activity confirmed complex V deficiency.

## 1 INTRODUCTION

Mitochondrial ATP synthase, a key enzyme of the mitochondrial energy provision, catalyzes the ATP synthesis during the oxidative phosphorylation (OXPHOS). Variants in the transmembrane protein 70 (*TMEM70*) gene (MIM number: 614052, mode of inheritance: autosomal recessive) are the leading cause of nuclear‐encoded ATP synthase deficiency, resulting in a syndrome characterized by a neonatal mitochondrial disorder with muscular hypotonia, cardiomyopathy, and apneic spells within hours after birth, variable central nervous system involvement accompanied by severe lactic acidosis, 3‐methylglutaconic aciduria (3‐MGA), and hyperammonemia^1^; however, the phenotype might markedly differ. Most patients are from a common Roma (ethnic) origin and homozygous for a single founder splice acceptor variant.^2^ Here we report the novel Japanese cases of *TMEM70* gene variants caused by compound heterozygous variants in a Japanese patient (c.141delG, p.Pro48Argfs*2, and c.316+1G>A; reference sequence GenBank NM_017866.5).

## 2 CASE

**Figure 1:**
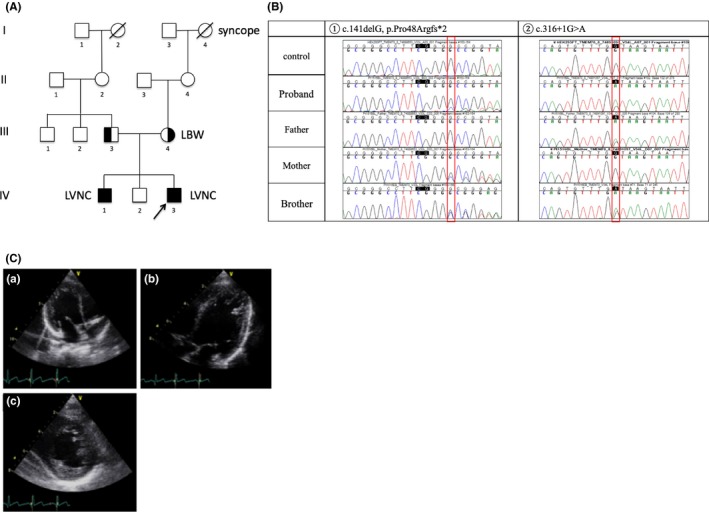
A, The family pedigree. B, The results of Sanger sequence of target alleles. C, An ultrasound image showing an abnormal, highly trabeculated left ventricular myocardium; four‐chamber view (a), long‐axis view (b), and short‐axis view (c) demonstrating prominent trabeculations and thick noncompacted layer in the LV and the two‐layered structure of noncompacted (NC) and compacted (C) layers NC/C > 2.0

A 2460‐g male newborn of Japanese parents born at 36 weeks of gestation with Apgar scores of 9/9 presented with mild tachypnea and poor sucking at 1 day and was referred to the Neonatal Intensive Care Unit at the Ishikawa Prefectural Central Hospital. We detected hyperlactacidemia (169.5 mg/dL), metabolic acidosis (pH 7.098, ABE, −22.1), and hyperalaninemia (229 μmol/L). The patient was treated for hypoglycemia (36 mg/dL) with hyperketonemia (1090 μmol/L), metabolic acidosis, hyperammonemia (229 μg/dL), and hyperlactacidemia. His face showed no dysmorphic features, and head ultrasonography revealed no abnormal findings. Echocardiography revealed mild left ventricular hypertrophy with a left ventricular ejection fraction of 70%. His therapy was initiated with infusion comprising 10% glucose and lipid emulsion with l‐carnitine, coenzyme Q10, and riboflavin. Hyperammonemia was treated with sodium phenylbutyrate. At the age of 40 days, the patient was discharged. Follow‐up echocardiography at 7 months of age revealed prominent trabeculation of the left ventricle with an inguinal hernia and undescended testicle, and the patient was referred to Toyama University Hospital at the age of 9 months. Echocardiography revealed prominent trabeculation and blood signals within the intertrabecular region (Figure 1). In addition, the urinary organic acids profile revealed increased levels of 3‐methylglutaconic acid. An investigation of family history revealed that the oldest brother had left ventricular noncompaction with mild mental and motor developmental delay, and the second oldest brother had polymicrogyria, with no further cases of cardiomyopathy, encephalopathy, or sudden death (Figure 1). At present, the patient is 2 years old and has a mild motor delay with mild short stature (−1.0 SD) and light body weight height (−1.0 SD), and his left ventricular ejection fraction is retained, but trabeculation is apparent.

We performed targeted gene panel of nuclear genes and mitochondrial genes that are listed in a previous publication^3^ using DNA extracted from blood samples obtained from the family with GAIIx and MiSeq (Illumina, San Diego, CA) paired‐end reads.^4^ Notably, this patient was one of those whose targeted gene panel was sequenced previously using a detailed protocol, including variant calling.^5^ The targeted gene panel sequencing detected no previously reported pathogenic variants that were considered as causative for cardiomyopathy. We used the primers designed in a previous study to assess the heteroplasmic status.^6^ In the patient and his oldest brother, we detected compound heterozygous variants in the *TMEM70* gene—deletion variant (NM_017866: c.141delG, p.Pro48Argfs*2) and splice site variant (c.316+1G>A) 7 (Figure 1). His father carried a deletion variant (c.141delG), whereas his mother carried a splice site variant (c.316+1G>A). Genetic testing was not performed because we did not get the consent from the parents due to social circumstances. Both variants are expected to result in the loss of *TMEM70* function but are extremely rare in the population (Table 1).

**Table 1:** Variants identified in the patients

NM_017866	NP_060336	dbSNP	ExAC	3.5KJPN
c.141delG	p.Pro48Argfs*2	NA	NA	NA
c.316+1G>A	‐	rs201401841	8.26E‐06	NA

**Figure 2:**
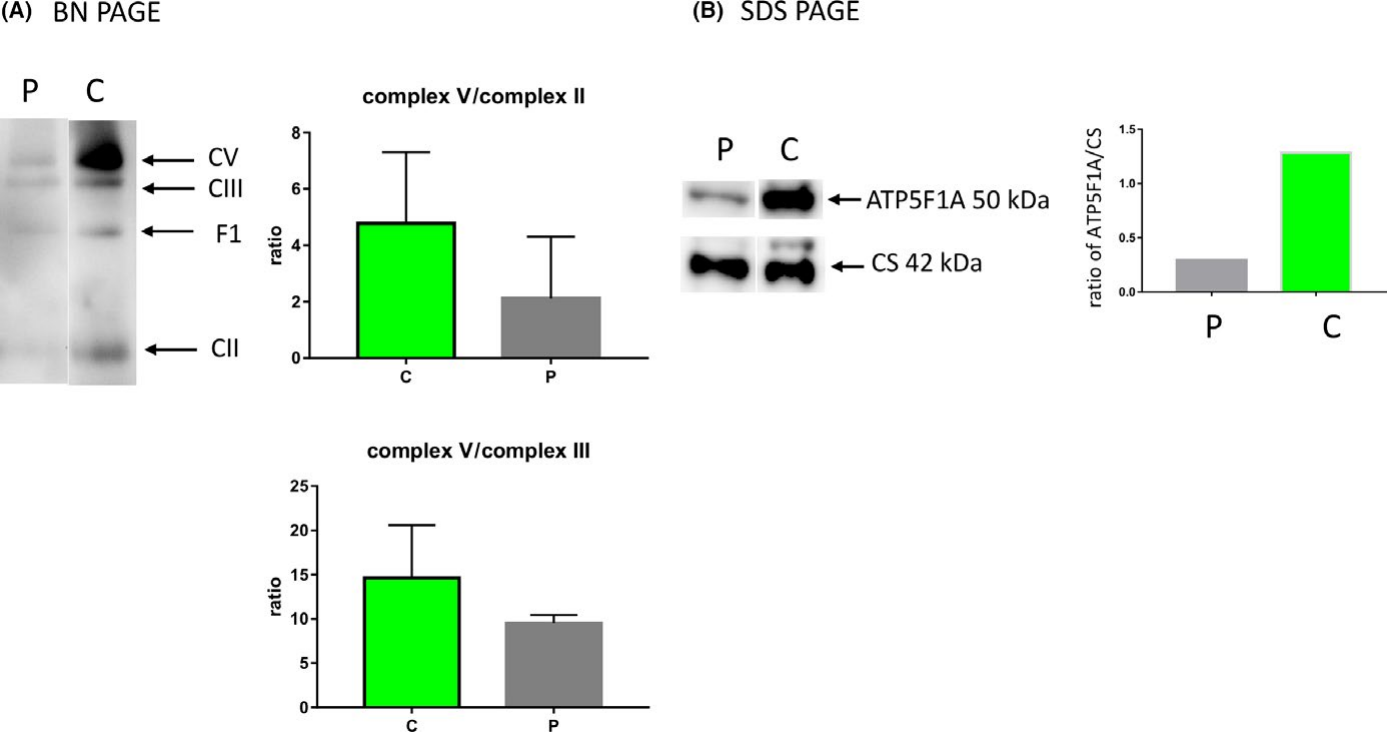
The electrophoretic analysis of the mitochondria from cultured fibroblasts. A, the mitochondria prepared from cultured skin fibroblasts were solubilized by the addition of 10% dodecylmaltoside at a protein concentration of 2 mg/mL; 15 μg of solubilized protein were loaded on a BN‐PAGE gel. This gel blotted to a PVDF membrane and was analyzed by western blotting. Arrows, the mobility of individual OXPHOS complexes. C, control; P, patient. B, proteins of the mitochondria (5 μg) from cultured fibroblasts were separated on SDS‐PAGE and analyzed by Western blotting using antibodies against citrate synthase (CS; molecular weight, 42 kDa) and against ATP synthase subunit F1‐α (msATP5F1A)

At the age of 2 years, the patient underwent skin biopsies. Blue native polyacrylamide gel electrophoresis (BN‐PAGE)/Western blotting determined complex V deficiency, and sodium dodecyl sulfate polyacrylamide gel electrophoresis (SDS‐PAGE)/Western blotting determined ATP synthase F1 subunit alpha protein levels, as previously described 8 (Figure 2). In addition, BN‐PAGE/Western blotting was used to detect the amounts of other OXPHOS complexes and revealed a reduction in complexes V compared with complexes II and III. Furthermore, SDS‐PAGE/Western blotting revealed the declined protein level of complex V (50 kDa of ATP5F1A).

Using a coupled colorimetric assay, we assessed the hydrolytic oligomycin‐sensitive ATPase activity of the ATP synthase following the decline of NADH at 340 nm, as reported previously.^9^ The ATP synthase activity significantly decreased (8 mU/mg protein; normal range: 43‐190 mU/mg protein), and the ratio of complex V/citrate synthase was low (0.134; normal range: 0.27‐0.69) in the skin fibroblasts of the patient. Finally, BN‐PAGE/Western blotting analysis and respiratory chain activity definitively diagnosed complex V deficiency.

Informed consent was obtained from all participants, according to the institutional guidelines. This study protocol conforms to the ethical guidelines of the 1975 Declaration of Helsinki as reflected in a priori approval by the Research Ethics Committee of University of Toyama, Japan.

## 3 DISCUSSION

To the best of our knowledge, this is the first report to present a Japanese patient with biallelic variants of the *TMEM70* gene. The patient presented with elevated plasma lactate levels and 3‐methylglutaconic acid in urine, undescended testicle, and infantile cardiomyopathy along with left ventricular noncompaction.

*TMEM70* is an essential factor in the biogenesis and stabilization of ATP synthase.^10^, ^11^ The mammalian OXPHOS comprises five multi‐subunit complexes, three of which, complexes I, III, and IV, support in the generation of a proton gradient across the inner mitochondrial membrane. Complex V, also named ATP synthase, transfers protons back to the inner mitochondrial membrane for the conversion of ADP and inorganic phosphate to ATP.^12^, ^13^ Complex V comprises two functional domains and is assembled of 16 subunits^14^; of these, two subunits are encoded by the mitochondrial DNA (*MT‐ATP6* and *MT‐ATP8*), and the remaining 14 subunits are encoded by nuclear DNA. Seemingly, complex V plays a vital role in the mitochondrial morphology.^15^ Several complex V‐related disorders are known, including mitochondrial and nuclear gene defects. Defects of nuclear genes, such as *ATPAF2*,* ATP5F1E*,* ATP5F1A*,* ATP5F1D*,* TMEM70*, and *USMG5*, result in complex V deficiency. Previously, variants in the *TMEM70* gene have been described in a group of patients sharing a similar biochemical defect and clinical phenotype with neonatal onset, characterized by lactic acidosis, cardiomyopathy (89%), 3‐MGA, and variable central nervous system involvement such as developmental delay (98%) and hypotonia (95%), which have been well characterized since the gene description in 2008 and 2015.^1^, ^7^ However, the following symptoms were further reported: faltering growth (94%), short stature (89%), microcephaly (71%), facial dysmorphism (66%), hypospadias (50% of the males), persistent pulmonary hypertension of the newborn (22%), and Wolff‐Parkinson‐White syndrome (13%).^7^ These symptoms were not observed in this patient.

Several lines of evidence support the pathogenic role of novel compound heterozygous variants. First, variants were found to segregate with the disease in the patient; the healthy father and mother were heterozygous for one of the *TMEM70* variants. Second, the frame‐shift variant was neither present in ExAC nor 3.5KJPN databases (http://exac.broadinstitute.org and https://ijgvd.megabank.tohoku.ac.jp). In addition, the ATP synthase activity was markedly decreased, and the ratio of complex V/citrate synthase was low in the skin fibroblasts of the patient, clearly suggesting complex V deficiency. The finding of the compound heterozygous variants in our patient confirm that the variants are pan‐ethnic and might also occur in genetic backgrounds other than the ethnic group of Roma.^16^

In conclusion, this report presents our findings in a patient with isolated complex V defect caused by two different variants in the *TMEM70* gene in Asian populations. As some of the pathogenic variants in the *TMEM70* gene were reported to be population‐specific, the prevalence of the *TMEM70* variants should be assessed in infantile cardiomyopathy patients in various populations.^16^ This report further expands the clinical and genetic spectrum of *TMEM70* deficiency. The development and progress of the new techniques of next‐generation sequencing will allow effective diagnosis, thereby avoiding invasive and unnecessary procedures and speeding up the diagnostic process. The identification of the potential candidate gene, together with the resulting more precise clinical and genomic characterization and clarification of genotype‐phenotype correlations, will allow the individualization of the follow‐up (in this case cardiologic) with potential improvement of the outcome. Nevertheless, further studies are warranted to confirm the relevance of these observations.

## CONFLICT OF INTEREST

None declared.

## AUTHOR CONTRIBUTIONS

KH and KM: conceived of the presented idea and wrote the manuscript with support from RGF and JAM. NN: collected the data. MOT, TF, MK, YK, YO: carried out the experiment. FI and AO: supervised the project.
